# Improving Prognostic Prediction in Head and Neck Cancer Through a Combined Systemic Immune-Inflammation Index and Prognostic Nutritional Index Score

**DOI:** 10.3390/curroncol33010030

**Published:** 2026-01-05

**Authors:** Takuya Miura, Hisashi Kessoku, Masato Nagaoka, Yohei Morishita, Toshiki Kobayashi, Hiromi Kojima

**Affiliations:** 1 Department of Otorhinolaryngology and Head and Neck Surgery, Jikei University Kashiwa Hospital, Kashiwa 277-8567, Japan; h-kessoku@jikei.ac.jp (H.K.); g.ymorishita1208@hotmail.co.jp (Y.M.); toshiki-koba@jikei.ac.jp (T.K.); 2 Department of Otorhinolaryngology, Jikei University Hospital, Tokyo 105-0003, Japan; maabou1208@yahoo.co.jp (M.N.); kojimah@jikei.ac.jp (H.K.)

**Keywords:** systemic immune–inflammation index, prognostic nutritional index, head and neck cancer

## Abstract

This study evaluated whether a composite index combining the systemic immune–inflammation index (SII) and prognostic nutritional index (PNI), the coSII–PNI score, enhances prognostic prediction in head and neck cancer. We retrospectively evaluated 166 patients who underwent curative surgery between 2015 and 2023. Patients were stratified into three groups according to the coSII–PNI score (range, 0–2) derived from preoperative blood data. The optimal cutoff values for SII and PNI were 743 and 49, respectively. A significant correlation was observed between the SII and PNI (r = −0.386, *p* < 0.01). Patients with a high coSII–PNI score (low SII + high PNI) showed significantly better disease-free and overall survival than those with lower scores (both *p* < 0.01). The areas under the curve for predicting prognosis were 0.649 for SII, 0.717 for PNI, and 0.730 for the coSII–PNI score. These findings indicate that integrating systemic inflammation and nutritional status improves prognostic accuracy compared with either index alone. Therefore, the coSII–PNI score may serve as a simple, practical preoperative biomarker for risk stratification in patients with head and neck cancer.

## Simple Summary

Head and neck cancer is often treated with surgery, but some patients still relapse or die even after treatment that is meant to cure them. Surgeons need a simple way to spot patients at higher risk before the operation so that they can provide extra support, such as nutritional care and closer follow-up. We looked at two measures that can be taken from routine blood tests before surgery: one that reflects levels of inflammation in the body and another that reflects nutritional and immune health. We combined these two measures into a single score and tested it in patients with head and neck cancer who had surgery at our hospital. Patients with an unfavorable score were more likely to die or have the cancer return. This simple blood test may help doctors identify susceptible patients early and better plan care and follow-up.

## 1. Introduction

In 2020, head and neck cancers (HNC) accounted for 4.5% of all cancer-related deaths worldwide, with approximately 450,000 fatalities [1]. The oral cavity is the most common tumor site, followed by the larynx, nasopharynx, oropharynx, and hypopharynx [1,2]. More than 90% of these cancers are squamous cell carcinomas [2]. Despite advances in treatment options, including surgery, radiation therapy, and chemoradiation [2], long-term outcomes remain suboptimal [2,3]. Thus, reliable biomarkers for prognostic prediction before treatment initiation are urgently required.

Systemic inflammation significantly influences tumor growth, advancement, invasion, and metastasis [4]. The systemic immune-inflammation index (SII), derived from peripheral blood neutrophil, platelet, and lymphocyte counts, is an emerging inflammatory marker that effectively predicts the prognosis of various cancers [5,6,7]. Malnutrition is associated with disease progression and shorter survival time [8,9]. In HNC, dysphagia and odynophagia reduce oral intake, while inflammation-driven hypermetabolism can contribute to deterioration of nutritional status [10,11]. Consequently, 30–50% of patients with HNC present with malnutrition [12,13]. The prognostic nutritional index (PNI) is a nutritional assessment tool calculated using serum albumin and lymphocyte counts. It has been validated as a reliable prognostic indicator for various diseases, including cancer [14,15].

In HNC, pretreatment SII and PNI levels have been shown to be significantly correlated with posttreatment outcomes [16,17,18]. Furthermore, combining PNI with other established prognostic factors has been shown to improve outcome prediction markedly [19,20,21]. The preoperative combination of SII and PNI has enhanced the accuracy of prognostic assessments in other cancers [20,21]. However, although the SII and PNI are individually associated with outcomes in HNC, direct evidence for a simple preoperative composite of these two indices is limited. In this study, we evaluated the prognostic relevance of a preoperative composite score integrating the SII and PNI (coSII–PNI score) in patients with HNC who underwent curative surgery at our institution.

## 2. Materials and Methods

### 2.1. Study Design and Population

This retrospective study included patients diagnosed with head and neck squamous cell carcinoma who underwent surgical treatment at the Department of Otorhinolaryngology and Head and Neck Surgery at Jikei University Kashiwa Hospital between January 2015 and December 2023. Patients who received postoperative chemotherapy or radiotherapy were excluded to minimize treatment-related confounding factors in survival analyses. A total of 166 patients were included in this analysis. Baseline patient information (age at first visit, sex), disease specifics (tumor stage [reassessed for pre-2018 cases using the 8th Edition of the Union for International Cancer Control TNM classification], details of curative treatment, and disease outcomes), and blood test data within 6 weeks before surgery (including complete blood count and albumin [Alb]) were collected for analysis. This study complied with the Strengthening the Reporting of Observational Studies in Epidemiology (STROBE) guidelines [22].

### 2.2. Index Score Calculation

SII was calculated as (platelet count × neutrophil count) ÷ lymphocyte count, and PNI was calculated as 10 × serum albumin (g/dL) + 0.005 × lymphocyte count (/mm^3^) [5,23]. We then defined a composite preoperative index, termed the coSII–PNI score (range, 0–2). Patients were divided into three groups according to the following criteria: group 0, high SII and low PNI; group 1, high SII and high PNI or low SII and low PNI; and group 2, low SII and high PNI. The coSII–PNI score was evaluated for its association with overall survival (OS) and disease-free survival (DFS).

### 2.3. Statistical Analysis

OS was defined as the time from surgery to any-cause death, and survivors were censored at their last contact. DFS was defined as the time from surgery to the first documented recurrence (local, regional, or distant) or death, whichever occurred first. Patients without events were censored at the last disease assessment. Statistical comparisons were performed using the Mann–Whitney U test for continuous variables and the Fisher exact test for categorical variables, and hazard ratios (HRs) with 95% confidence intervals (95% CIs) were reported. The optimal SII and PNI cutoffs were determined by calculating the Youden index from the receiver operating characteristic (ROC) curve, and the discriminatory ability of SII, PNI, and the coSII–PNI score for predicting 5-year OS was assessed using ROC curves and the area under the curve (AUC). DFS and OS were estimated using the Kaplan–Meier method, and survival differences between the groups were evaluated using the log-rank test. Associations between patient and disease characteristics, including SII and PNI, and prognosis were examined using univariate Cox proportional hazards models; significant factors were included as covariates in multivariate Cox models. The Spearman rank correlation analysis was additionally conducted to assess the relationship between SII and PNI. All statistical analyses were performed using Stata/SE 19.5 (StataCorp LLC., College Station, TX, USA). Analyses were performed using complete cases. Continuous variables are presented as mean ± standard deviation (SD) or median (range), and categorical variables are summarized as counts and percentages. All tests were two-sided, and statistical significance was set at *p* < 0.05.

## 3. Results

### 3.1. Patient Characteristics

The median patient age was 69 years (range, 39–89 years), and there were 141 men and 25 women. The primary tumor sites were the oral cavity (*n* = 61), oropharynx (*n* = 34), hypopharynx (*n* = 43), and larynx (*n* = 28). In total, 31, 28, 44, and 63 patients had clinical stages I, II, III, and IV disease, respectively. All 166 patients underwent curative surgery; 113 underwent reconstructive procedures, while 53 did not. The mean interval between blood testing and surgery was 20.6 ± 9.1 days. At the last follow-up, 13 patients experienced local recurrence, and 22 developed distant metastasis. Patient characteristics are shown in Table 1.

### 3.2. Optimal Cutoff Values and Group Comparisons

**Figure 1:**
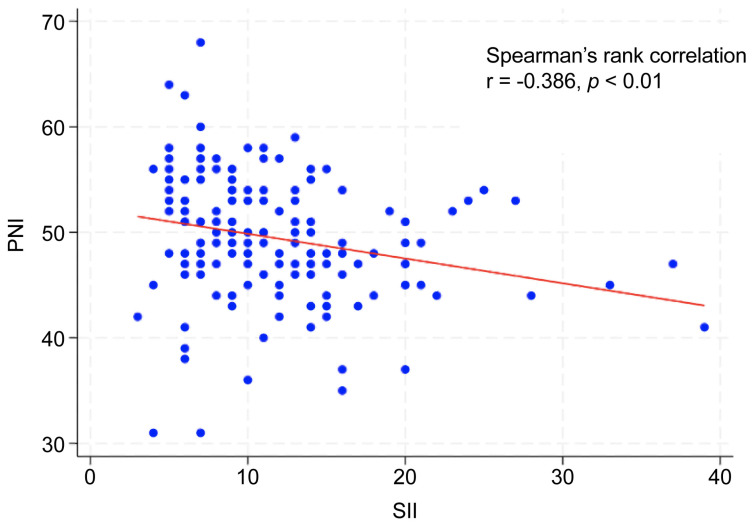
Correlation between SII and PNI.

The optimal cutoff values were 743 for SII and 49 for PNI (Table 1). The AUC for predicting 5-year OS was 0.649 (95% CI: 0.546–0.751) for SII, with a Youden index of 0.324, sensitivity of 0.615, and specificity of 0.708 (*p* < 0.001). The AUC of PNI was 0.717 (95% CI: 0.628–0.805), with a Youden index of 0.334, sensitivity of 0.744, and specificity of 0.591 (*p* < 0.01). When patients were stratified into two groups (high vs. low) according to each cutoff value, the clinical stage and primary tumor site (oral cavity and hypopharynx) differed significantly between the high- and low-SII groups. Age, sex, clinical stage, and primary tumor site (oropharynx, hypopharynx, and larynx) differed significantly between the high and low PNI groups (Table 2). Additionally, Spearman’s rank correlation analysis revealed a significant negative correlation between the SII and PNI (r = −0.386, *p* < 0.01; Figure 1).

### 3.3. Survival Outcomes

**Figure 2:**
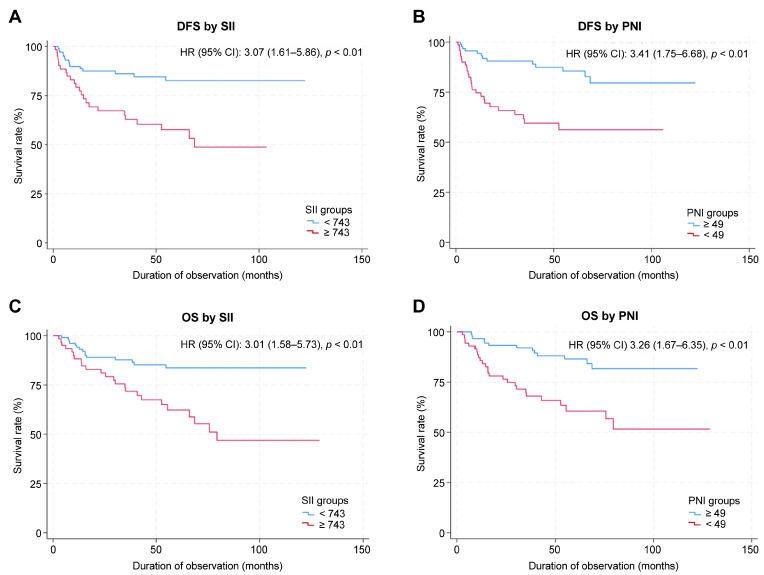
Kaplan–Meier survival curves for SII and PNI. (**A**) DFS by SII. (**B**) DFS by PNI. (**C**) OS by SII. (**D**) OS by PNI.

The median follow-up duration was 63.3 months (95% CI: 56.0–68.7 months). The 5-year OS rate for the entire cohort was 75.7% (95% CI: 67.6–82.0%), and the 5-year DFS rate was 73.1% (95% CI: 64.5–79.9%). When stratified by SII score, the 5-year OS was 81.6% (95% CI: 72.2–88.2%) in the low SII group and 63.4% (95% CI: 48.8–74.8%) in the high SII group, respectively. The 5-year DFS rate was 82.6% (95% CI: 72.4–89.3%) in the low SII group and 57.6% (95% CI: 42.6–70.1%) in the high SII group. When stratified by PNI score, the 5-year OS rate was 64.3% (95% CI: 48.7–76.2%) in the low PNI group and 84.6% (95% CI: 74.7–90.8%) in the high-PNI group. The 5-year DFS was 56.3% (95% CI: 41.8–68.4%) in the low-PNI group and 85.6% (95% CI: 75.2–91.9%) in the high-PNI group. The Kaplan–Meier survival curves for OS and DFS, stratified by SII and PNI cutoff values, are shown in Figure 2. The log-rank test revealed significant between-group differences in both OS and DFS (*p* < 0.01; Figure 2).

### 3.4. Prognostic Factors for DFS and OS

Univariate analysis identified significant associations between DFS and clinical stage (HR: 12.84, *p* < 0.001), primary tumor site (hypopharynx: HR, 8.28; *p* = 0.005; larynx: HR, 8.13; *p* = 0.006, relative to oropharynx), SII (HR: 3.07, *p* = 0.001), and PNI (HR: 3.41, *p* < 0.001). Multivariate analysis identified advanced clinical stage and high SII as independent factors associated with shorter DFS (Table 3).

Univariate analysis identified significant associations between OS and age (HR, 1.03; *p* = 0.04), clinical stage (HR, 11.25; *p* = 0.001), primary tumor site (hypopharynx: HR, 7.56; *p* = 0.01; larynx: HR, 8.31, *p* = 0.01; relative to the oropharynx), SII (HR, 3.01; *p* = 0.001), and PNI (HR, 3.26; *p* = 0.001). Multivariate analysis showed that the clinical stage and high SII were independently associated with shorter OS (Table 4).

### 3.5. Prognostic Utility of the coSII–PNI Score

**Figure 3:**
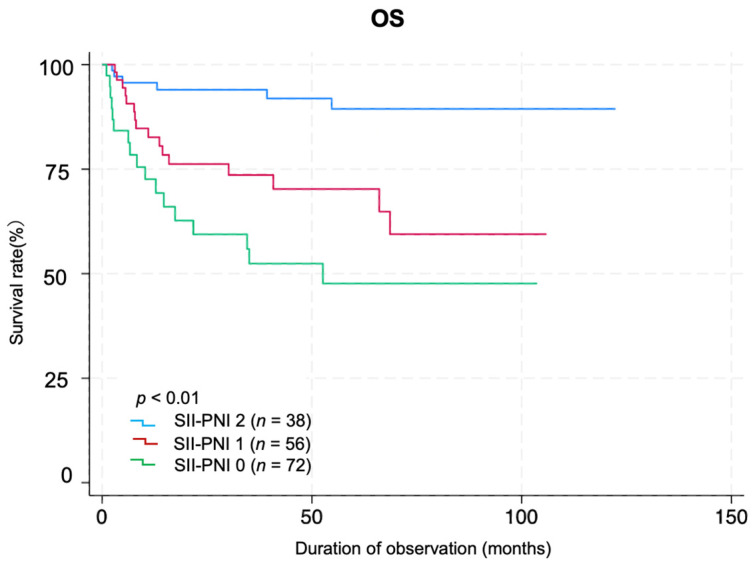
Kaplan–Meier survival curves by coSII–PNI score group. Patients were divided into three groups according to the coSII–PNI score: score 0, high SII + low PNI (*n* = 72); score 1, high SII + high PNI or low SII + low PNI (*n* = 56); and score 2, low SII + high PNI (*n* = 38). Kaplan–Meier survival curves are shown for each group, and the log-rank test shows significant differences between the groups (*p* < 0.01).

**Figure 4:**
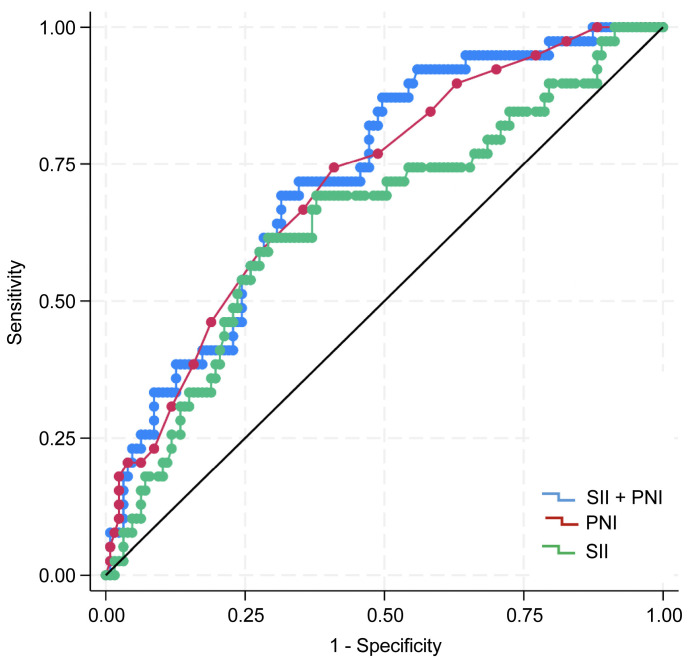
ROC curves of the SII, PNI, and coSII–PNI score for predicting 5-year overall survival (OS). The AUC of the SII was 0.649 (95% confidence interval [CI]: 0.546–0.751), the Youden index was 0.324; sensitivity was 0.615; and specificity was 0.708 (*p* < 0.001). The AUC of PNI was 0.717 (95% CI: 0.628–0.805), the Youden index was 0.334; sensitivity was 0.744; and specificity was 0.591 (*p* < 0.01). The AUC of the coSII–PNI score was 0.730 (95% CI: 0.644–0.815), with a Youden index of 0.371; sensitivity of 0.718; and specificity of 0.654 (*p* < 0.01). AUC, area under the curve; CI, confidence interval.

A total of 72, 56, and 38 patients belonged to groups 0, 1, and 2, respectively, based on the coSII–PNI score. Log-rank analysis of Kaplan–Meier survival curves revealed significant differences among the groups (*p* < 0.01). Group 0 had the worst prognoses (Figure 3). The AUC value of SII for predicting prognosis was 0.649 (95% CI: 0.546–0.751), with a Youden index of 0.324, sensitivity of 0.615, and specificity of 0.708 (*p* < 0.001). The AUC of PNI was 0.717 (95% CI: 0.628–0.805), with a Youden index of 0.334, sensitivity of 0.744, and specificity of 0.591 (*p* < 0.01). The coSII–PNI score had the highest AUC for predicting prognosis at 0.730 (95% CI: 0.644–0.815), with a Youden index of 0.371, sensitivity of 0.718, and specificity of 0.654 (*p* < 0.01) (Figure 4).

## 4. Discussion

In patients with HNC, even curative surgery does not eliminate the risk of recurrence or death, and clinicians currently lack a simple preoperative tool to identify patients at the highest risk. In this study, we found that a combined preoperative score reflecting systemic inflammation and nutritional/immune status was strongly associated with oncologic outcomes. Patients with unfavorable scores (high inflammation and poor nutritional/immune status) were more likely to experience recurrence and had shorter OS than those with favorable scores. This two-component blood-based score also discriminated prognosis better than either marker alone, suggesting that it may help stratify surgical candidates and guide perioperative optimization in the future.

Systemic inflammation and poor nutritional status are associated with an unfavorable prognosis in various cancers [24,25,26]. In 2014, Hu et al. introduced the SII as a measure of systemic inflammation to predict postoperative outcomes in patients with hepatocellular carcinoma [5]. Using neutrophil, lymphocyte, and platelet counts from peripheral blood, the SII provides a simple, objective method to gauge the inflammatory response. Its prognostic utility has been confirmed across multiple cancer types [7,27,28,29,30], and it outperforms traditional inflammatory markers in both inflammation and prognosis, suggesting its potential as a cornerstone of tailored therapeutic approaches [26,31]. The PNI was developed by Buzby et al. in 1980 to assess postoperative complication risk in patients with gastric cancer by assessing systemic nutritional status [32]. Subsequently, in 1984, Onodera et al. simplified the index by using only serum albumin and lymphocyte count. This simplified index has since become the most widely used in clinical oncology research [23]. The PNI comprehensively reflects nutritional and immune status, as determined by serum albumin and lymphocyte counts. Recent studies have established the prognostic significance of this index in various malignancies [33,34].

Previous studies have combined the systemic immune-inflammation index (SII) and prognostic nutritional index (PNI) to improve prognostic prediction. Ding et al. showed that a combined SII–PNI score effectively predicted treatment response and prognosis in patients with locally advanced gastric cancer who received preoperative chemoimmunotherapy with the PD-1 antibody sintilimab plus the XELOX regimen [35]. Similarly, Chen et al. found that the preoperative combined SII and PNI values were significantly associated with poor prognosis in patients with epithelial ovarian cancer who underwent surgery [36]. Furthermore, Zhang et al. reported, in a review of multiple solid tumors, that the combination of SII and PNI was useful for predicting survival outcomes [37].

Previous studies on HNC have demonstrated that both SII and PNI are valuable prognostic factors [38,39,40,41]. However, studies integrating these two indices are scarce. Wang et al. recently reported the potential of their novel systemic immune-inflammation-nutrition score, incorporating the SII, PNI, and other inflammation- and nutrition-related parameters, for individualized prognostic prediction for patients with HNC [42]. Although there have been increasing efforts to move from single markers to composite evaluations, studies combining the SII and PNI for prognostic assessment of HNC remain scarce. The present study addresses this research gap by developing the coSII–PNI score and demonstrating its clinical utility.

Our findings revealed a significant negative correlation between the SII and PNI in patients with HNC, suggesting that elevated systemic inflammation corresponds to diminished immune function and nutritional health. Additionally, low preoperative PNI and high SII were significantly associated with advanced clinical stage. These results highlight the SII and PNI as effective indicators of the body’s response to tumor progression, consistent with findings in other cancers, such as gastric and colorectal cancers [43,44]. Multivariate analysis indicated that the preoperative SII was an independent prognostic factor. Although PNI showed a significant association with outcomes in the univariate analyses, it was not an independent factor after adjustment. We developed the coSII–PNI score, a composite index that integrates the SII and PNI. Patients with a high coSII–PNI score (score 2: low SII + high PNI) had the most favorable prognosis, whereas those with a low coSII–PNI score (score 0: high SII + low PNI) had the poorest prognoses.

The following biological mechanisms may underlie the association between the coSII and PNI scores and prognosis. Patients with a low coSII–PNI score have relatively greater numbers of neutrophils and platelets than lymphocytes, which may contribute to an immunosuppressive state or promote tumor progression [45]. Neutrophils suppress the cytotoxic activity of lymphocyte-activated killer cells, thereby reducing the host antitumor cellular immune response [46,47]. Additionally, neutrophils release vascular endothelial growth factor, which promotes tumor angiogenesis, invasion, and metastasis [48]. Platelets release cytokines such as platelet-derived growth factor and fibroblast growth factor, which are implicated in tumor growth and vascular metastasis [49,50]. A decrease in lymphocyte count reflects a weakened antitumor immune response, further facilitating tumor progression [51,52]. Moreover, low serum albumin levels indicate malnutrition, which contributes to tumor progression and poor prognosis by impairing the immune function [53,54].

In this study, patients with high PNI and low SII had the best prognosis, whereas those with low PNI and high SII had the worst. The coSII–PNI score had a higher AUC for predicting prognosis than either SII or PNI alone, indicating improved prognostic accuracy. These findings support the notion that a favorable nutritional status and controlled inflammatory response before treatment may contribute to better outcomes. Furthermore, the results validated the hypothesis that combining the SII and PNI provides greater predictive accuracy than either index alone in patients with HNC. This finding aligns with observations in other cancers, such as ovarian and esophageal cancers, reported in previous studies [20,36]. Collectively, these findings offer significant insights into the utility of the coSII–PNI score as a comprehensive, objective prognostic indicator for assessing nutritional status and the inflammatory response in patients with HNC.

In recent years, the importance of individualized perioperative management strategies tailored to patient backgrounds and risks has grown, grounded in the core principles of Enhanced Recovery After Surgery (ERAS). Mithany et al. emphasized that ERAS components should be flexibly adjusted according to the patient’s condition [55]. Zhao et al. reported that personalized ERAS programs reduced postoperative complications [56]. Prospective interventional studies aimed at optimizing perioperative care for HNC are underway. These include an ongoing phase III randomized controlled trial in Japan (PreSte-HN Study) that evaluates the efficacy of preoperative steroid administration [57]. The coSII–PNI score in this study accurately reflected perioperative biological responses and showed potential as a stratification tool for determining the suitability of interventions such as preoperative steroid administration. Moving forward, the application of the coSII–PNI score in interventional studies and the implementation of individually optimized perioperative management are expected to facilitate the development of more effective, patient-centered treatment strategies.

This study has some limitations. This was a single-center retrospective study with a limited sample size, which may restrict the completeness and generalizability of the data. In addition, the optimal cutoff values for the SII and PNI have not yet been standardized, and their validity and reproducibility require confirmation in large-scale, multicenter prospective studies. We also aim to validate the coSII–PNI score in an independent future multicenter cohort. In addition to SII and PNI, other inflammation-based indices—such as the C-reactive protein/albumin ratio, the neutrophil-to-lymphocyte ratio, and the platelet-to-lymphocyte ratio—have been reported to be useful for prognostic stratification in HNC [58,59,60]. However, the present study did not evaluate these indices or other inflammation- and nutrition-related markers, including the Glasgow Prognostic Score, the lymphocyte-to-monocyte ratio, and the monocyte-to-lymphocyte ratio. Future studies directly comparing these indices may clarify their complementary roles and further improve prognostic stratification.

## 5. Conclusions

The preoperative coSII–PNI score is associated with the survival of patients with HNC. The coSII–PNI score provides more accurate prognostic discrimination than either index alone and thus better informs preoperative risk stratification. The coSII–PNI score may be used to stratify patients in future trials evaluating nutritional support and inflammation control.

## Figures and Tables

**Table 1:** Baseline patient characteristics (*n* = 166).

Characteristic	Value
Age, years	
Median (range)	69 (39–89)
Sex, *n* (%)	
Male	141 (84.9)
Female	25 (15.1)
Clinical stage, *n* (%)	
I	31 (18.7)
II	28 (16.9)
III	44 (26.5)
IVa	62 (37.4)
IVb	1 (0.6)
Primary tumor site, *n* (%)	
Oral cavity	61 (36.8)
Oropharynx	34 (20.5)
Hypopharynx	43 (26.0)
Larynx	28 (16.9)
Reconstructive surgery, *n* (%)	
Yes	113 (68.1)
No	53 (31.9)
Lymphocyte count,/mm^3^	
Median (range)	1700 (500–4300)
Neutrophil count,/mm^3^	
Median (range)	4300 (1500–11,900)
Platelet count,/mm^3^ × 10^3^	
Median (range)	243 (105–566)
Serum albumin concentration, g/dL	
Median (range)	4.1 (2.0–5.1)
SII	
Cutoff value (range)	743 (172–2822)
PNI	
Cutoff value (range)	49 (31–68)

**Table 2:** Comparison of baseline patient data across low and high SII and low and high PNI groups.

Variable	SII, *n* (%)			PNI, *n* (%)		
	<743	≥743	*p* Value	<49	≥49	*p* Value
Age (years)						
<69	53 (67.1)	26 (32.9)	0.339	27 (34.1)	52 (65.8)	0.041 *
≥69	52 (59.8)	35 (40.2)		44 (50.6)	43 (49.4)	
Sex						
Male	89 (63.1)	52 (36.9)	1.000	67 (47.5)	74 (52.5)	0.004 *
Female	16 (64.0)	9 (36.0)		4 (16.0)	21 (84.0)	
Clinical stage						
I–II	46 (78.0)	13 (22.0)	0.004 *	12 (20.3)	47 (79.7)	<0.001 *
III–IV	59 (55.1)	48 (44.9)		59 (55.1)	48 (44.9)	
Primary tumor site						
Oral cavity	45 (73.8)	16 (26.2)	0.045 *	21 (34.4)	40 (65.6)	0.106
Other than the oral cavity	60 (57.1)	45 (42.9)		50 (47.6)	55 (52.3)	
Oropharynx	23 (67.6)	11 (32.4)	0.690	7 (20.6)	27 (79.4)	0.003 *
Other than the oropharynx	82 (62.1)	50 (37.9)		64 (48.5)	68 (51.5)	
Hypopharynx	20 (46.5)	23 (53.5)	0.010 *	25 (58.1)	18 (41.9)	0.021 *
Other than the hypopharynx	85 (69.1)	38 (30.9)		46 (37.4)	77 (62.6)	
Larynx	17 (60.7)	11 (39.3)	0.831	18 (64.3)	10 (35.7)	0.020 *
Other than the larynx	88 (63.8)	50 (36.2)		53 (38.4)	85 (63.4)	
Reconstructive surgery						
Yes	68 (60.2)	45 (39.8)	0.300	48 (42.5)	65 (57.5)	1.000
No	37 (69.8)	16 (30.2)		23 (43.4)	30 (56.6)	

* Statistically significant, *p* < 0.05.

**Table 3:** Univariate and multivariate analyses of the factors influencing disease-free survival.

Variable	Univariate Analysis		Multivariate Analysis	
	Hazard Ratio (95% CI)	*p* Value	Hazard Ratio (95% CI)	*p* Value
Age	1.03 (1.00–1.06)	0.082		
Sex				
Female	Reference	-		
Male	2.21 (0.68–7.19)	0.186		
Clinical stage				
I–II	Reference	-	Reference	
III–IV	12.84 (2.39–41.77)	<0.001 *	8.02 (1.81–35.58)	0.006 *
Primary tumor site				
Oropharynx	Reference	-	Reference	
Oral cavity	3.31 (0.72–15.11)	0.122	3.21 (0.70–14.72)	0.134
Hypopharynx	8.27 (1.87–107.14)	0.005 *	3.23 (0.72–14.38)	0.125
Larynx	8.12 (1.80–36.68)	0.006 *	3.99 (0.87–18.41)	0.076
Reconstructive surgery				
No	Reference	-		
Yes	1.13 (0.56–2.30)	0.715		
SII				
<743	Reference	-	Reference	-
≥743	3.07 (1.61–5.86)	0.001 *	2.33 (1.19–4.58)	0.014 *
PNI				
≥49	Reference	-	Reference	-
<49	3.41 (1.75–6.67)	<0.001 *	1.97 (0.99–3.89)	0.052

The multivariable Cox proportional hazards model included clinical stage and primary tumor site as covariates. * Statistical significance (*p* < 0.05).

**Table 4:** Univariate and multivariate analyses of the factors influencing overall survival.

Variable	Univariate Analysis		Multivariate Analysis	
	Hazard Ratio (95% CI)	*p* Value	Hazard Ratio (95% CI)	*p* Value
Age	1.03 (1.00–1.07)	0.040 *	1.02 (0.98–1.05)	0.405
Sex				
Female	Reference	-		
Male	2.08 (0.64–7.62)	0.223		
Clinical stage				
I–II	Reference	-	Reference	
III–IV	11.25 (2.71–46.70)	0.001 *	6.34 (1.43–28.13)	0.015 *
Primary tumor site				
Oropharynx	Reference	-	Reference	
Oral cavity	2.99 (0.65–13.68)	0.157	2.47 (0.54–11.38)	0.247
Hypopharynx	7.56 (1.74–32.88)	0.007 *	2.90 (0.64–13.16)	0.167
Larynx	8.31 (1.84–37.58)	0.006 *	3.43 (0.72–16.40)	0.122
Reconstructive surgery				
No	Reference	-		
Yes	0.88 (0.44–1.77)	0.715		
SII				
<743	Reference	-	Reference	-
≥743	3.01 (1.57–5.73)	0.001 *	2.29 (1.18–4.44)	0.014 *
PNI				
≥49	Reference	-	Reference	-
<49	3.26 (1.67–6.35)	0.001 *	1.85 (0.93–3.69)	0.081

The multivariable Cox proportional hazards model included age, clinical stage, and primary tumor site as covariates. * Statistically significant, *p* < 0.05.

## Data Availability

The data supporting this study’s findings were derived from institutional clinical records and included potentially identifiable patient information. Therefore, the datasets are not publicly available because of institutional and ethical restrictions. De-identified data may be made available from the corresponding author upon reasonable request and with the permission of the Ethics Committee of the Jikei University School of Medicine.

## Abbreviations

The following abbreviations are used in this manuscript:

AUC	area under the curve
CI	confidence interval
DFS	disease-free survival
ERAS	Enhanced Recovery After Surgery
HR	hazard ratio
HNC	head and neck cancer
OS	overall survival
PNI	prognostic nutritional index
ROC	receiver operating characteristic
SII	systemic immune–inflammation index

## References

[1] Sung H., Ferlay J., Siegel R.L., Laversanne M., Soerjomataram I., Jemal A., Bray F. (2021). Global cancer statistics 2020: GLOBOCAN estimates of incidence and mortality worldwide for 36 cancers in 185 countries. CA Cancer J. Clin..

[2] Johnson D.E., Burtness B., Leemans C.R., Lui V.W.Y., Bauman J.E., Grandis J.R. (2020). Head and neck squamous cell carcinoma. Nat. Rev. Dis. Primers.

[3] Patterson R.H., Fischman V.G., Wasserman I., Siu J., Shrime M.G., Fagan J.J., Koch W., Alkire B.C. (2020). Global burden of head and neck cancer: Economic consequences, health, and the role of surgery. Otolaryngol. Head Neck Surg..

[4] Proctor M.J., McMillan D.C., Morrison D.S., Fletcher C.D., Horgan P.G., Clarke S.J. (2012). A derived neutrophil to lymphocyte ratio predicts survival in patients with cancer. Br. J. Cancer.

[5] Hu B., Yang X.R., Xu Y., Sun Y.F., Sun C., Guo W., Zhang X., Wang W.M., Qiu S.J., Zhou J. (2014). Systemic immune-inflammation index predicts prognosis of patients after curative resection for hepatocellular carcinoma. Clin. Cancer Res..

[6] Wang Q., Zhu D. (2019). The prognostic value of systemic immune-inflammation index (SII) in patients after radical operation for carcinoma of stomach in gastric cancer. J. Gastrointest. Oncol..

[7] Guo W., Cai S., Zhang F., Shao F., Zhang G., Zhou Y., Zhao L., Tan F., Gao S., He J. (2019). Systemic immune-inflammation index (SII) is useful to predict survival outcomes in patients with surgically resected non-small cell lung cancer. Thorac. Cancer.

[8] Engelstrup E., Beck A.M., Munk T., Bardal P., Knudsen A.W. (2023). The association between nutrition impact symptoms, nutritional risk, and risk of reduced overall survival in patients with head and neck cancer. A retrospective study. Clin. Nutr. ESPEN.

[9] Chang P.H., Hsieh J.C.H., Yeh K.Y., Chen E.Y.C., Yang S.W., Huang J.S., Lai C.H., Wu T.H., Huang Y.M., Chang Y.S. (2018). Prognostic nutritional index relevance in chemoradiotherapy for advanced oral cavity, oropharyngeal and hypopharyngeal cancer. Asia Pac. J. Clin. Nutr..

[10] Ravasco P., Monteiro-Grillo I., Vidal P.M., Camilo M.E. (2003). Nutritional deterioration in cancer: The role of disease and diet. Clin. Oncol. (R. Coll. Radiol.).

[11] de Carvalho T.M.R., Miguel Marin D.M., da Silva C.A., de Souza A.L., Talamoni M., Lima C.S.P., Monte Alegre S. (2015). Evaluation of patients with head and neck cancer performing standard treatment in relation to body composition, resting metabolic rate, and inflammatory cytokines. Head Neck.

[12] Vílchez-López F.J., González-Pacheco M., Fernández-Jiménez R., Zarco-Martín M.T., Gonzalo-Marín M., Cobo-Molinos J., Carmona-Llanos A., Muñoz-Garach A., García-Luna P.P., Herrera-Martínez A.D. (2024). Predictive factors of the degrees of malnutrition according to GLIM criteria in head and neck cancer patients: Valor group. Cancers.

[13] Kono T., Sakamoto K., Shinden S., Ogawa K. (2017). Pre-therapeutic nutritional assessment for predicting severe adverse events in patients with head and neck cancer treated by radiotherapy. Clin. Nutr..

[14] Chen Q.J., Qu H.J., Li D.Z., Li X.M., Zhu J.J., Xiang Y., Li L., Ma Y.T., Yang Y.N. (2017). Prognostic nutritional index predicts clinical outcome in patients with acute ST-segment elevation myocardial infarction undergoing primary percutaneous coronary intervention. Sci. Rep..

[15] Correa-Rodríguez M., Pocovi-Gerardino G., Callejas-Rubio J.L., Fernández R.R., Martín-Amada M., Cruz-Caparros M.G., Ortego-Centeno N., Rueda-Medina B. (2019). The prognostic nutritional index and nutritional risk index are associated with disease activity in patients with systemic lupus erythematosus. Nutrients.

[16] Wang Y.T., Kuo L.T., Weng H.H., Hsu C.M., Tsai M.S., Chang G.H., Lee Y.C., Huang E.I., Tsai Y.T. (2022). Systemic immune-inflammation index as a predictor for head and neck cancer prognosis: A meta-analysis. Front. Oncol..

[17] Wang E.Y., Chen M.K., Hsieh M.Y., Kor C.T., Liu Y.T. (2022). Relationship between preoperative nutritional status and clinical outcomes in patients with head and neck cancer. Nutrients.

[18] Luan C.W., Tsai Y.T., Yang H.Y., Chen K.Y., Chen P.H., Chou H.H. (2021). Pretreatment prognostic nutritional index as a prognostic marker in head and neck cancer: A systematic review and meta-analysis. Sci. Rep..

[19] Uri I., Horváth A., Tamás L., Polony G., Dános K. (2024). Prognostic nutritional index (PNI) correlates with survival in head and neck cancer patients more precisely than other nutritional markers—Real world data. Eur. Arch. Otorhinolaryngol..

[20] Zhang H., Shang X., Ren P., Gong L., Ahmed A., Ma Z., Ma R., Wu X., Xiao X., Jiang H. (2019). The predictive value of a preoperative systemic immune-inflammation index and prognostic nutritional index in patients with esophageal squamous cell carcinoma. J. Cell Physiol..

[21] He H., Guo W., Song P., Liu L., Zhang G., Wang Y., Qiu B., Tan F., Xue Q., Gao S. (2020). Preoperative systemic immune-inflammation index and prognostic nutritional index predict prognosis of patients with pulmonary neuroendocrine tumors after surgical resection. Ann. Transl. Med..

[22] von Elm E., Altman D.G., Egger M., Pocock S.J., Gøtzsche P.C., Vandenbroucke J.P., STROBE Initiative (2007). The Strengthening the Reporting of Observational Studies in Epidemiology (STROBE) statement: Guidelines for reporting observational studies. Ann. Intern. Med..

[23] Onodera T., Goseki N., Kosaki G. (1984). Prognostic nutritional index in gastrointestinal surgery of malnourished cancer patients. Nihon Geka Gakkai Zasshi.

[24] Yan X., Zhu J., Wang J., Lu Y., Ye X., Sun X., Jiang H., Li Z., He C., Zhai W. (2024). Development and validation of a novel prognostic prediction system based on GLIM-defined malnutrition for colorectal cancer patients post-radical surgery. Front. Nutr..

[25] Zheng P., Wang B., Luo Y., Duan R., Feng T. (2024). Research progress on predictive models for malnutrition in cancer patients. Front. Nutr..

[26] Zhong J.-H., Huang D.-H., Chen Z.-Y. (2017). Prognostic role of systemic immune-inflammation index in solid tumors: A systematic review and meta-analysis. Oncotarget.

[27] Yang R., Chang Q., Meng X., Gao N., Wang W. (2018). Prognostic value of systemic immune-inflammation index in cancer: A meta-analysis. J. Cancer.

[28] Chen J.H., Zhai E.T., Yuan Y.J., Wu K.M., Xu J.B., Peng J.J., Chen C.Q., He Y.L., Cai S.R. (2017). Systemic immune-inflammation index for predicting prognosis of colorectal cancer. World J. Gastroenterol..

[29] Lolli C., Caffo O., Scarpi E., Aieta M., Conteduca V., Maines F., Bianchi E., Massari F., Veccia A., Chiuri V.E. (2016). Systemic immune-inflammation index predicts the clinical outcome in patients with mCRPC treated with abiraterone. Front. Pharmacol..

[30] Deng C., Zhang N., Wang Y., Jiang S., Lu M., Huang Y., Ma J., Hu C., Hou T. (2019). High systemic immune-inflammation index predicts poor prognosis in advanced lung adenocarcinoma patients treated with EGFR-TKIs. Medicine.

[31] Geng Y., Shao Y., Zhu D., Zheng X., Zhou Q., Zhou W., Ni X., Wu C., Jiang J. (2016). Systemic immune-inflammation index predicts prognosis of patients with esophageal squamous cell carcinoma: A propensity score-matched analysis. Sci. Rep..

[32] Buzby G.P., Mullen J.L., Matthews D.C., Hobbs C.L., Rosato E.F. (1980). Prognostic nutritional index in gastrointestinal surgery. Am. J. Surg..

[33] Okadome K., Baba Y., Yagi T., Kiyozumi Y., Ishimoto T., Iwatsuki M., Miyamoto Y., Yoshida N., Watanabe M., Baba H. (2020). Prognostic nutritional index, Tumor-infiltrating Lymphocytes, and Prognosis in Patients with Esophageal Cancer. Ann. Surg..

[34] Maejima K., Taniai N., Yoshida H. (2022). The prognostic nutritional index as a predictor of gastric cancer progression and recurrence. J. Nippon. Med. Sch..

[35] Ding P., Guo H., Sun C., Yang P., Kim N.H., Tian Y., Liu Y., Liu P., Li Y., Zhao Q. (2022). Combined systemic immune-inflammatory index (SII) and prognostic nutritional index (PNI) predicts chemotherapy response and prognosis in locally advanced gastric cancer patients receiving neoadjuvant chemotherapy with PD-1 antibody sintilimab and XELOX: A prospective study. BMC Gastroenterol..

[36] Chen J., Jin L., Luo R., Zhang X., Chen Y., Han Z., Liu T. (2025). Predictive value of preoperative systemic immune-inflammation index and prognostic nutrition index in patients with epithelial ovarian cancer. J. Ovarian Res..

[37] Zhang Y., Tang M., Gu Q.-H., Zhou L.-N., Chen M.-B. (2025). The prognostic value of combined systemic immune-inflammatory index (SII) and prognostic nutritional index (PNI) in solid tumor. Cancer Manag. Res..

[38] Xu J., Lin Y., Yang J., Xing Y., Xing X. (2024). Pretreatment systemic immune-inflammation index and lymphocyte-to-monocyte ratio as prognostic factors in oral cavity cancer: A meta-analysis. Medicine.

[39] Ye M., Zhang L. (2024). Correlation of prognostic nutritional index and systemic immune-inflammation index with the recurrence and prognosis in oral squamous cell carcinoma with the stage of III/IV. Int. J. Gen. Med..

[40] Kubota K., Ito R., Narita N., Tanaka Y., Furudate K., Akiyama N., Chih C.H., Komatsu S., Kobayashi W. (2022). Utility of prognostic nutritional index and systemic immune-inflammation index in oral cancer treatment. BMC Cancer.

[41] Atasever Akkas E.A., Erdis E., Yucel B. (2023). Prognostic value of the systemic immune-inflammation index, systemic inflammation response index, and prognostic nutritional index in head and neck cancer. Eur. Arch. Otorhinolaryngol..

[42] Wang W.Y., Chen Y., Chen Q., Sun H.W., Niu N.X., Li H.H., Cao Y.D., Bai Y.X., Li X. (2024). Nomogram-derived immune-inflammation-nutrition score could act as a novel prognostic indicator for patients with head and neck squamous cell carcinoma. Front. Immunol..

[43] Lee J.Y., Kim H.I., Kim Y.N., Hong J.H., Alshomimi S., An J.Y., Cheong J.H., Hyung W.J., Noh S.H., Kim C.B. (2016). Clinical significance of the prognostic nutritional index for predicting short- and long-term surgical outcomes after gastrectomy: A retrospective analysis of 7781 gastric cancer patients. Medicine.

[44] Tan Y., Hu B., Li Q., Cao W. (2025). Prognostic value and clinicopathological significance of pre-and post-treatment systemic immune-inflammation index in colorectal cancer patients: A meta-analysis. World J. Surg. Oncol..

[45] Houghton A.M., Rzymkiewicz D.M., Ji H., Gregory A.D., Egea E.E., Metz H.E., Stolz D.B., Land S.R., Marconcini L.A., Kliment C.R. (2010). Neutrophil elastase-mediated degradation of IRS-1 accelerates lung tumor growth. Nat. Med..

[46] Siwicki M., Pittet M.J. (2021). Versatile neutrophil functions in cancer. Semin. Immunol..

[47] Shau H.Y., Golub S.H. (1989). Inhibition of lymphokine-activated killer- and natural killer-mediated cytotoxicities by neutrophils. J. Immunol..

[48] Gong Y., Koh D.R. (2010). Neutrophils promote inflammatory angiogenesis via release of preformed VEGF in an in vivo corneal model. Cell Tissue Res..

[49] Pintucci G., Froum S., Pinnell J., Mignatti P., Rafii S., Green D. (2002). Trophic effects of platelets on cultured endothelial cells are mediated by platelet-associated fibroblast growth factor-2 (FGF-2) and vascular endothelial growth factor (VEGF). Thromb. Haemost..

[50] Verheul H.M., Jorna A.S., Hoekman K., Broxterman H.J., Gebbink M.F., Pinedo H.M. (2000). Vascular endothelial growth factor–stimulated endothelial cells promote adhesion and activation of platelets. Blood.

[51] Whiteside T.L. (2013). Immune modulation of T-cell and NK (natural killer) cell activities by TEXs (tumour-derived exosomes). Biochem. Soc. Trans..

[52] Feng X.Y., Wen X.Z., Tan X.J., Hou J.H., Ding Y., Wang K.F., Dong J., Zhou Z.W., Chen Y.B., Zhang X.S. (2015). Ectopic expression of B and T lymphocyte attenuator in gastric cancer: A potential independent prognostic factor in patients with gastric cancer. Mol. Med. Rep..

[53] Migita K., Matsumoto S., Wakatsuki K., Kunishige T., Nakade H., Miyao S., Sho M. (2021). Effect of oral nutritional supplementation on the prognostic nutritional index in gastric cancer patients. Nutr. Cancer.

[54] Kitayama J., Yasuda K., Kawai K., Sunami E., Nagawa H. (2010). Circulating lymphocyte number has a positive association with tumor response in neoadjuvant chemoradiotherapy for advanced rectal cancer. Radiat. Oncol..

[55] Mithany R.H., Daniel N., Shahid M.H., Aslam S., Abdelmaseeh M., Gerges F., Gill M.U., Abdallah S.B., Hannan A., Saeed M.T. (2023). Revolutionizing surgical care: The power of enhanced recovery after surgery (ERAS). Cureus.

[56] Zhao X., Jin S., Peng M., Wang J. (2024). A retrospective study on the efficacy of the ERAS protocol in patients who underwent laparoscopic left and right colectomy surgeries. Front. Surg..

[57] Shinozaki T., Imai T., Kobayashi K., Yoshimoto S., Zenda S., Yamaguchi T., Eguchi K., Okano T., Mashiko T., Kurosaki M. (2023). Preoperative steroid for enhancing patients’ recovery after head and neck cancer surgery with free tissue transfer reconstruction: Protocol for a phase III, placebo-controlled, randomised, double-blind study (J-SUPPORT 2022, PreSte-HN Study). BMJ Open.

[58] Mascarella M.A., Mannard E., Silva S.D., Zeitouni A. (2018). Neutrophil-to-lymphocyte ratio in head and neck cancer prognosis: A systematic review and meta-analysis. Head Neck.

[59] Takenaka Y., Oya R., Kitamiura T., Ashida N., Shimizu K., Takemura K., Yamamoto Y., Uno A. (2018). Platelet count and platelet–lymphocyte ratio as prognostic markers for head and neck squamous cell carcinoma: Meta-analysis. Head Neck.

[60] Luan C.-W., Yang H.-Y., Tsai Y.-T., Hsieh M.-C., Chou H.-H., Chen K.-S. (2021). Prognostic value of C-reactive protein-to-albumin ratio in head and neck cancer: A meta-analysis. Diagnostics.

